# Expression and Regulation of Thymic Stromal Lymphopoietin and Thymic Stromal Lymphopoietin Receptor Heterocomplex in the Innate–Adaptive Immunity of Pediatric Asthma

**DOI:** 10.3390/ijms19041231

**Published:** 2018-04-18

**Authors:** Sheng-Chieh Lin, Fang-Yi Cheng, Jun-Jen Liu, Yi-Ling Ye

**Affiliations:** 1 Department of Pediatrics, Shuang Ho Hospital, Taipei Medical University, Taipei 23561, Taiwan; jacklinmails@yahoo.com.tw; 2 Department of Pediatrics, School of Medicine, College of Medicine, Taipei Medical University, Taipei 11031, Taiwan; 3 Graduate Institute of Clinical Medicine, College of Medicine, National Taiwan University, Taipei 10002, Taiwan; 4 Department of Clinical Pathology, Far Eastern Memorial Hospital, New Taipei City 22060, Taiwan; fangyicheng7261@gmail.com; 5 School of Medical Laboratory Science and Biotechnology, Taipei Medical University, Taipei 11031, Taiwan; jjliu_96@tmu.edu.tw; 6 Ph.D. Program in Biotechnology Research and Development, College of Pharmacy, Taipei Medical University, Taipei 11031, Taiwan; 7 Department of Biotechnology, National Formosa University, Yunlin County 63201, Taiwan

**Keywords:** IL-7Rα, pediatric asthma, thymic stromal lymphopoietin, thymic stromal lymphopoietin receptor

## Abstract

Asthma is a chronic inflammatory disease affecting the airway, and it is characterized by a wheezing breathing sound, variable airflow obstruction and the presence of inflammatory cells in the submucosa of the bronchi. Viral infection, pollutants and sensitivity to aeroallergens damage the epithelium from childhood, which causes asthma. The pathogenesis of asthma includes pathways of innate stimulation by environmental microbes and irritant pathogens. Damaged epithelial cells produce thymic stromal lymphopoietin (TSLP) and stimulate myeloid dendritic cell maturation through the thymic stromal lymphopoietin receptor (TSLPR) heterocomplex. TSLP-activated myeloid dendritic cells promote naive CD4^+^ T cells to differentiate into T helper type 2 (Th2) phenotype CD4^+^ T cells. Re-exposure to allergens or environmental stimuli causes an adaptive immune response. TSLP-activated dendritic cells expressing the OX40 ligand (OX40L; CD252) trigger naive CD4^+^ T cells to differentiate into inflammatory Th2 effector cells secreting the cytokines interleukin-4, 5, 9, and 13 (IL-4, IL-5, IL-9 and IL-13), and the dendritic cells (DCs) promote the proliferation of allergen-specific Th2 memory cells. Allergen presentation by Th2 cells through its interaction with their receptors in the presence of major histocompatibility complex (MHC) class II on B cells and through costimulation involving CD40 and CD40L interactions results in immunoglobulin class switching from IgM to IgE. DCs and other blood cell subsets express the TSLPR heterocomplex. The regulatory mechanism of the TSLPR heterocomplex on these different cell subsets remains unclear. The TSLPR heterocomplex is composed of the IL-7Rα chain and TSLPR chain. Moreover, two isoforms of TSLP, short isoform TSLP (sfTSLP) and long isoform TSLP (lfTSLP), have roles in atopic and allergic development. Identifying and clarifying the regulation of TSLPR and IL-7Rα in pediatric asthma are still difficult, because the type of blood cell and the expression for each blood cell in different stages of atopic diseases are poorly understood. We believe that further integrated assessments of the regulation mechanism of the TSLP–TSLPR heterocomplex axis in vitro and in vivo can provide a faster and earlier diagnosis of pediatric asthma and promote the development of more effective preventive strategies at the onset of allergies.

## 1. Introduction

**Figure 1:**
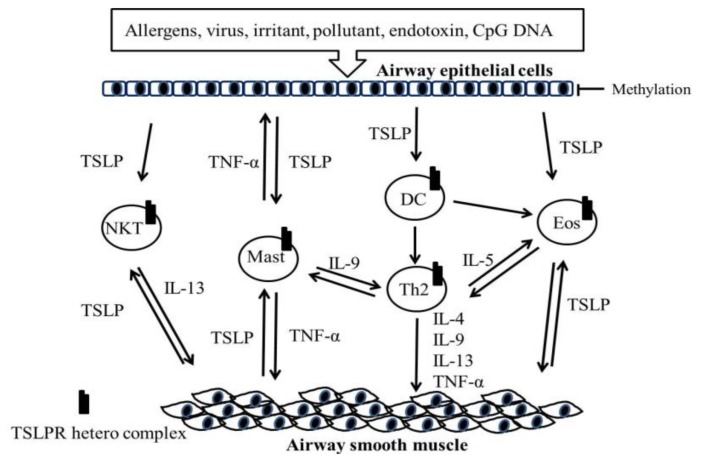
Role of TSLP in asthma. Airway epithelial-secreted TSLP after stimulation by allergens, viruses, irritants, pollutants, endotoxins and CpG DNA. TSLP can activate dendritic cells, mast cells, NKT cells and eosinophils to interact with cytokines and inflammatory mediators on the airway smooth muscle of patients with asthma. TSLP: thymic stromal lymphopoietin; TNF-α: tumor necrosis factor-α; NKT: natural killer T cells; Mast: mast cells; DC: dendritic cell; Eos: eosinophils; IL-4: interleukin-4; IL-9: interleukin-9; IL-13: interleukin-13.

Asthma is a chronic inflammatory respiratory disease leading to variable airflow obstruction. Many immune cells, including dendrite cells, T cells, B cells, eosinophils, basophils and mast cells, infiltrate into the submucosa of bronchi and cause a series of immune reactions in patients with asthma [1]. The presence of inflammatory cells in the airway causes an altered repair response and the secretion of growth factors, which induce structural changes in the airway, termed airway remodeling [2,3]. Remodeling includes mucus cell metaplasia, hyperplasia and hypertrophy of the airway smooth muscle, angiogenesis, fibrosis and the increase of inflammatory cells [4,5]. Dendritic cells (DCs) located in the airway epithelium and underlying mucosa are a type of antigen-presenting cell. These cells express receptors of the innate immune system and take up allergens to process them into small peptides, presenting them through the major histocompatibility complex (MHC) classes I and II for recognition by T cell receptors [6]. Airways do not contain DCs at birth. Microbe and irritant damage to the activation of the respiratory epithelium are probably the main innate immunologic stimuli initiating the ingression of immature DCs from the bone marrow [6,7]. Such damage and activation also cause the release of chemoattractants such as chemokine (C-C motif) ligand 20, 19, and 27 (CCL20, CCL19 and CCL27) and the ligands for C-C chemokine receptor type 6, 7, and 10 (CCR6, CCR7 and CCR10), thus directing DC migration toward the epithelium and underlying mucosa [6,7]. Damaged epithelial cells can produce thymic stromal lymphopoietin (TSLP), thus stimulating myeloid DC maturation [6]. TSLP also activates DCs through TSLPR and promotes DCs to cause the differentiation of naive CD4^+^ T cells into the Th2 phenotype [8]. TSLP can also directly activate mast cells after the stimulation of epithelial cells and can induce mast cells to release multiple proinflammatory cytokines and chemokines independent of immunoglobulin E (IgE) [9]. Additionally, activated IgE-mediated mast cells can release tumor necrosis factor-α (TNF-α), which may induce smooth muscle cells to produce TSLP from inside the airway [9]. TSLP also upregulates interleukin-13 (IL-13) production in natural killer T cells and decreases airway hyper-reactivity in an asthma model [10]. TSLP can stimulate human eosinophils through the activation of extracellular signal-regulated protein kinase, p38 mitogen-activated protein kinase and the nuclear factor-κB (NF-κB)-dependent signaling pathway [10]. Re-exposure to allergens or environmental stimuli can cause an adaptive immune response. In patients with asthma, exposure to allergens (such as pollen, mold spores, dust mites, animal dander and dust), viruses or environmental stimuli can cause adaptive immune responses. TSLP-activated DCs expressing the OX40 ligand (OX40L; CD252) can trigger naive CD4^+^ T cells to differentiate into inflammatory T helper type 2 (Th2) cells and the expansion of allergen-specific Th2 memory cells [11]. Inflammatory Th2 effector cells also secrete the cytokines of IL-4, IL-9 and IL-13, which enhance IgE, mast cell and mucous production and increase airway hyper-responsiveness. In allergen-specific Th2 memory cells, the MHC class II-associated allergen interacts with the receptors on B cells and costimulates CD40 and CD40L interactions, resulting in immunoglobulin class switching from IgM to IgE [6]. This results in the selective expansion of T lymphocytes (particularly the Th2 type), which secrete cytokines encoded on chromosome 5q31–33, including interleukins IL-3, IL-4, IL-5, IL-9 and IL-13, and the granulocyte macrophage colony-stimulating factor (GM-CSF) that causes airway smooth muscle contraction and vasodilatation and increased vascular permeability and mucous secretion [1,6]. Lloyd and Saglani [12] indicated that T cells influence the pathway of asthma by reacting to the genetic and environmental exposures and interacting with structural cells such as epithelial cells, thus influencing inflammation. A study revealed that the mechanisms of pulmonary viral clearance might trigger innate and adaptive immune responses in patients with asthma [13]. Toll-like receptors (TLRs) also have critical effects on the innate immune system. Viral double- and single-stranded RNA, endotoxin and bacterial CpGoligodeoxynucleotides (CpG)-containing DNA activate selective TLRs on epithelial cells, enhancing DCs motility and antigen processing through the cytokines of TSLP that result in Th2 maturation [6,8]. TSLP is a key cytokine that initiates the DC-mediated Th2 immune response [14]. The TSLP–TSLPR heterocomplex axis may play a fundamental role in the innate–adaptive interface in the pathology of asthma (Figure 1).

## 2. Pediatric Asthma

Viral infection, environment, allergens, genetics, nutrition and immune responses play crucial roles in pediatric asthma [15,16]. However, the precise mechanisms of pediatric asthma remain unclear. In a Danish study, Harpsoe et al. [17] reported that maternal obesity and gestational weight gain increase the risk of asthma in children. A rapid increase in the body weight index during the first two years of childhood up to age of six results in an increased risk of pediatric asthma [18]. Viral infections are responsible for a substantial proportion of instances of asthma exacerbation in young children [19]. Cysteinyl leukotriene levels are elevated in children with asthma and are also increased during respiratory syncytial virus bronchiolitis, which causes acute bronchiolitis leading to the development of pediatric asthma [20,21]. Sex, urbanization and geographic region are all significantly associated with acute bronchiolitis and pediatric asthma [19]. A previous study also revealed that human asthmatic epithelium cells produce higher TSLP levels in response to respiratory syncytial viral infections [22]. This may also explain why respiratory viral infections exacerbate the symptoms of bronchial asthma. Parainfluenza type 1 is the most common causative pathogen of croup. The adjusted hazard ratio for asthma was 1.78-times higher in children with croup living in urban areas than in those living in rural areas [23]. Viral infections that trigger TSLP secretion may increase the risk of asthma. Moreover, research on DNA methylation in pediatric asthma has been conducted. Reduced whole blood DNA methylation at 14 CpG sites associated with transcriptional profiles indicates the activation of eosinophils and cytotoxic T cells in pediatric asthma [24]. A clinical diagnosis of asthma is difficult if the patient is younger than two years. In Taiwan, the diagnosis of asthma is based on the Global Initiative for Asthma guidelines. In older children, spirometry with forced expiratory volume in 1 s (FEV_1_) less than 80% can assist in diagnosing pediatric asthma. The treatment response to bronchodilators reflects the reversibility of airway obstruction and can be used as an adjunctive test to diagnose pediatric asthma. The response to a short-acting bronchodilator can be expressed by the absolute change in FEV_1_ [25]. A change of 12% or higher and 200 mL or above in the FEV_1_ from the baseline is commonly defined as a significant response to treatment [26]. However, Tse et al. [25] examined the diagnostic accuracy of the bronchodilator response of 12% and concluded that a threshold of less than 8% is superior to 12% for asthma diagnosis. Malinovschi et al. [27] demonstrated that exhaled nitric oxide is an indicator of inflammation and is thus related to the diagnosis of asthma. Oh et al. [28] reported that exhaled nitric oxide might be a more favorable biomarker than airway hyper-responsiveness and pulmonary function for asthma phenotypes in preschool children. Epidemiological studies have indicated that the occurrence of suspended substances in the environment is strongly correlated with the development of asthma. When these substances are inhaled into the respiratory tract, they may cause allergies and inflammation of the airway. Therefore, the avoidance of allergens can prevent asthma development in children. Clinically, the main treatments for asthma can be divided into five broad categories: (1) steroids: corticosteroids [29], prednisone [30] and methylprednisolone (Solumedrol) [31]; (2) leukotriene modifiers: montelukast (Singulair); (3) theophylline (Xanthium) [32]; (4) bronchodilators salmeterol (Serevent) [33], albuterol (Ventolin) [34], bambuterol (Bambec) [35], fenoterol (Berotec) and terbutaline (Bricanyl); and (5) Intalinhaler: cromolyn sodium [36]. Corticosteroids, currently the most efficacious drugs used to treat asthma and respiratory irritation [29], inhibit proinflammatory protein production [37]; reduce the number of eosinophils, T lymphocytes, mast cells and DCs during respiratory inflammation [38]; and decrease the incidence of asthma and exercise-induced asthma [39]. However, they have numerous side effects, such as dependency on drug dosage [40]. Overuse of steroids may elicit side effects such as inhibition of growth hormone secretion [41] and development of osteoporosis [42], adrenal insufficiency [43] and diabetes [44]. Salmeterol is a long-acting β2-agonist drug that reduces the severity of asthma in children [45] and suppresses TSLP secretion in human bronchial epithelial cells [33]. Usually, for the acute exacerbation of asthma, bronchodilators, such as the short-acting β2-agonist, are the primary drugs administered to relive shortness of breath. During an asthma attack, children experience difficulty breathing, which may cause mortality. However, asthma attacks in children may not be due to exposure to allergens; cold air exposure and excessive exercise are also notable causes. Pediatric asthma attacks often have different triggers, but the clinical symptoms are similar. Approximately 5%–10% of those who experience an asthma attack have a history of severe asthma [19]. Usually, the condition of such patients cannot be effectively controlled. These patients are a high-risk group who are likely to die due to asthma. Although patients with asthma have different allergies, chronic bronchial inflammation and airway fibrosis are the main causes of asthma. Previous studies have revealed that certain genes regulate cytokines and antibodies in allergic diseases [46]. However, in some patients, asthma cannot be detected using an allergen-specific antibody. Additionally, some children are too young to undergo spirometry. Therefore, these children cannot be diagnosed early and may die due to an asthma attack. The early diagnosis of asthma in children is crucial. The TSLP–TSLPR heterocomplex axis is involved in innate–adaptive immunity; the TSLPR heterocomplex may be an early biomarker of the development of asthma and therefore warrants further investigation.

## 3. Thymic Stromal Lymphopoietin

From 25 genome-wide association studies of asthma, 16 genes including TSLP were implicated in disease pathophysiology, as indicated by functional studies [47]. TSLP has been identified in culture supernatants of murine thymic stromal cells and has been defined as an epithelial-derived cytokine. The human *TSLP* gene is located on chromosome 5q22.1 [48,49]. It is a four-helix bundle cytokine [50]. Its function is similar to that of IL-7, in that it can promote the early differentiation of T and B cells. It also promotes B cell proliferation and prevents cell apoptosis [49,51]. Many cells can produce TSLP, including epithelial cells and keratinocytes in the skin, gut, lung, eye tissue and thymus [52,53]. TSLP can also be secreted by mast cells [54], basophils and DCs [55]. In humans, atopic dermatitis is characterized by a high level of TSLP in skin lesions, which is secreted by keratinocytes [56]. Keratinocytes that lack retinoid X receptors produce TSLP to induce atopic dermatitis [57]. In patients with allergic conjunctivitis, conjunctival epithelial cells produce TSLP, which activates DCs and induces *IL-13* mRNA expression in mast cells synergistically with IL-33 to cause ocular allergy [58]. In patients with allergic rhinitis, TSLP expression is increased in the nasal mucosa and is strongly correlated with the number of eosinophils and the clinical severity of symptoms [59]. In patients with autoimmune diseases, TSLP may also trigger Th1 and Th17 to secret proinflammatory cytokines that contribute to tissue inflammation [60]. TSLP and its receptor play a proinflammatory role by enhancing Th17 cells and causing tissue destruction in patients with autoimmune arthritis [60]. Recently, researchers have revealed that the secretion of TSLP by cancer cells can reduce the antitumor activity of Th1 cells [61]. However, the role of TSLP in tumor progression is still controversial [62]. Recently, two isoforms of TSLP have been discovered. Increasing studies have focused on the role of these two isoforms in diseases. These isoforms consist of 159 amino acids and 60 amino acids, respectively [48,49]. The short isoform TSLP (sfTSLP) exerts homeostatic effects, whereas the long isoform TSLP (lfTSLP) exerts inflammatory effects [63,64]. lfTSLP can be regulated through TLR 2, 3, 5 and 6 to maintain Th2 cytokine secretion [53]. In human keratinocytes, toll-like receptor ligands (polyI:C, FSL-1 and flagellin), as well as proinflammatory cytokines (Interferon-γ (IFN-γ), TNF and IL-1β) are potent inducers of lfTSLP, but not sfTSLP [63,64,65]. In human intestinal and skin tissue, the ratio of these two isoforms is highly correlated with the severity of inflammatory disorders [66]. Additionally, 1α,25-dihydroxyvitamin D3 (1,25D3), sfTSLP inducer, and sfTSLP itself can alleviate house dust mite-induced asthmatic airway inflammation [67]. Because lfTSLP and sfTSLP have the same C-terminal portion, C-terminal targeting neutralization leads to severe side effects, because these two isoforms have opposite functions. The relationship between the polymorphisms of the *TSLP* gene promoter and susceptibility to bronchial asthma [33] has been evaluated using sex-stratified analysis method [68,69].

## 4. TSLPR Heterocomplex

Immune regulation disorder is a key factor in disease development in humans [70]. It is crucial to maintain the balance between TSLP secretion by the epithelium and TSLPR heterocomplex expression levels in target cells. In peripheral blood, the TSLPR heterocomplex is considerably expressed in monocytes [71], DCs [72], lymphocytes [73] eosinophils [10,74], basophils [75] and mast cells [76]. TSLPR heterocomplex is composed of TSLPR (also known as cytokine receptor-like factor 2 (CRLF2)) [77] and the IL-7Rα chain [78]. The regulation of these two subunits of the TSLPR heterocomplex is different. Exon 4 of *IL-7Rα* single-nucleotide polymorphism (SNP) is correlated with the severity of asthma [79,80]. After TSLP binds to the TSLPR heterocomplex, the phosphorylation of Janus kinase1 (Jak1) and 2 (Jak2) activates signal transducers and activators of transcription (STATs) proteins, including STAT1, STAT3, STAT4, STAT5a, STAT5b and STAT6. TSLP also activates other signaling molecules such as phosphatidylinositol-3-kinase (PI3K)-protein Kinase B (AKT)- mammalian target of rapamycin complex 1 (mTORC1) pathway (PI3K/Akt/mTOR pathway), Proto-oncogene tyrosine-protein kinase/Tyrosine-protein kinase pathway (SRC/TEC pathway), extracellular-signal-regulated kinase 1/2 (ERK1/2), NF-κB, c-Jun N-terminal kinase 1/2 (JNK1/2) and P38 mitogen-activated protein kinases (P38MAPK) activation [81,82]. Through the TSLPR heterocomplex, TSLP-induced bone marrow-derived DCs (mDCs) express higher levels of CD40, CD54, CD80 and CD86 surface markers and secrete IL-8, eotaxin, macrophage-derived chemokine (MDC) and thymus and activation regulated chemokine (TARC). Though CCR4 interaction, mDCs activate T cells polarized to Th2 cell development. Th2-type cell development leads T cells to secrete IL-4, IL-5, IL-13 and TNF. Simultaneously, the secretion of Th1-type cytokines such as IFNγ, IL-12, IL-23 and IL-27 is suppressed by treatment with TSLP [83,84]. TSLP and TSLPR heterocomplex cognation activates the Jak-STAT signaling pathway, which leads to the proliferation and chemotaxis of eosinophils. Although sfTSLP can bind to the TSLPR heterocomplex and has superior antimicrobial peptide (AMP) activity to lfTSLP, it regulates atopic inflammation, in contrast to traditional TSLP (lfTSLP). The receptor that mediates this effect is still unclear [64,66,85], as is the unique receptor of sfTSLP. Recently, structural interactions between TSLP, TSLPR and IL-7Rα have been reported [86]. The association of *IL-7Rα* gene polymorphisms with asthma has been observed in different populations [79,80,87]. *TSLPR* SNP analysis has been conducted in eosinophilic esophagitis [88], atopic dermatitis and eczema herpeticum [89], but the *TSLPR* SNP in pediatric asthma is still unclear. The administration of a TSLPR blocker can alleviate the severity of asthma [81,82,83,84,85,90]. Owing to the complicated regulation by the two isoforms of the TSLP and TSLP receptor heterocomplex, especially the expression of IL-7Rα is highly regulated and involved in T cell activation [91,92]. It is a considerable challenge in the therapeutic application of the TSLP blocker [86].

## 5. Role of the TSLP and TSLPR Heterocomplex in Asthma

Studies have evaluated the role of the TSLP and TSLPR heterocomplex in asthma. Epithelial cells, neutrophils, endothelial cells, macrophages and mast cells have been revealed to be significant sources of TSLP in asthmatic patients [93]. TSLP is associated with atopic diseases, and *TSLP* genes are associated with allergic inflammation mechanisms, including eosinophil levels, IgE levels and bronchial asthma [8]. TSLP is a signature “Th2-favoring” or proallergic cytokine that has recently been linked to asthma [82]. A high level of TSLP in asthmatic airways has been correlated with Th2-attracting chemokines and disease severity [93]. TSLP and TARC/CCL17 expression is associated with airway obstruction in patients with asthma [8,54]. TSLP-influenced pulmonary Treg function is associated with tolerogenic immunity and increased protein expression in bronchoalveolar lavage fluids in the airways of patients with asthma [94]. Moreover, human asthmatic epithelial cells express TSLPR heterocomplex, and TSLP induces IL-13 production, which increases bronchial epithelial cell proliferation and injury repair [95]. The viral and genetic risk factors for the TSLP and TSLPR heterocomplex may have important roles in the onset of asthma. In response to rhinovirus infection, asthmatic epithelial cells produce higher pro-Th2 cytokine TSLP and lower Interferon-β (IFN-β) levels [96]. A previous study revealed that compared with the population without asthma, the SNPs of the *TSLPR* gene had a higher allele frequency of 33G > C in patients with asthma [97]. However, the polymorphisms of TSLPR were not significantly associated with total IgE, forced vital capacity (FVC) or FEV_1_ in patients with asthma [79]. A recent study reported that epistasis between *TSLP* and *SPINK5* genes contributes to pediatric asthma [98]. Additionally, TSLP assists natural helper cells to induce corticosteroid resistance in patients with asthma [99]. The anti-TSLP antibody reduces allergen-induced bronchoconstriction in patients with allergen-induced asthma [100]. It also decreases sputum and blood eosinophils in patients with allergic asthma [100]. The human anti-TSLP antibody named tezepelumab in the phase 2 study had a reduced annualized rate of asthma attack on uncontrolled asthmatic adults that were already treated with medium-to-high doses of inhaled glucocorticoids and long-acting β-agonists. [101].

## 6. Conclusions

**Figure 2:**
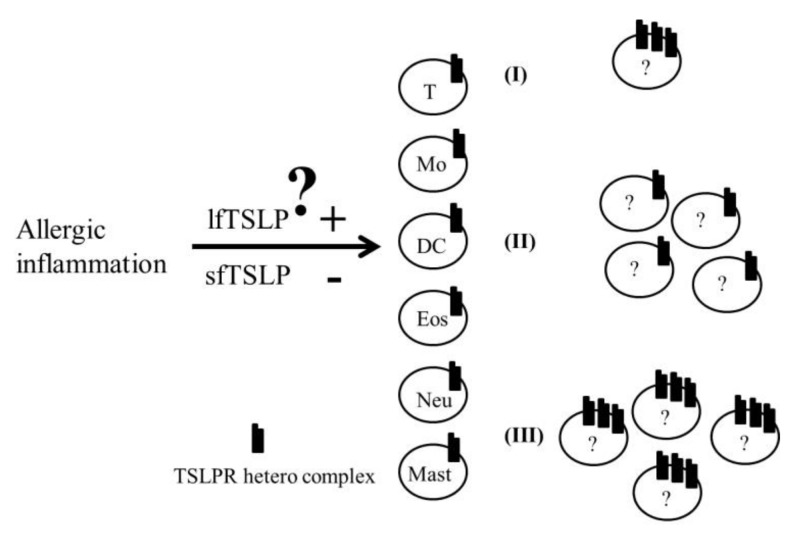
Hypothesis of TSLPR heterocomplex regulation during allergic inflammation. Three possible expression changes occur after allergic inflammation: (I) TSLPR heterocomplex expression increased on the cell membrane surface by one or several specific cell subsets. (II) TSLPR heterocomplex expression by one or several specific cell membrane surfaces does not change, but the absolute number of one or several specific cell subsets is increased. (III) A combination of the phenomenain (I) and (II). “?” means cell subsets from T, Mo: monocyte; DC: dendritic cell; Eos: eosinophil; Neu: neutrophil; or mast cells; lfTSLP: long isoform of TSLP; sfTSLP: short isoform.

Atopic diseases such as asthma, allergic rhinitis and atopic dermatitis are Th2-dominated inflammatory diseases Genetic, environmental and nutritional factors all contribute to the development of allergies, and more effective diagnostic markers are urgently required [102]. The role of the TSLP–TSLPR heterocomplex axis in these atopic diseases is increasingly pertinent and requires clarification [53]. Additionally, in clinical settings, the diagnosis of pediatric atopic asthma requires many criteria, clinical symptoms, family history and other biochemical analyses such as total IgE, allergen-specific IgE and eosinophil cell count, to be fulfilled. Additionally, lung function must be evaluated, and practitioners should directly assess discomfort in patients. Asthmatic symptom scoring, spirometry and inhaled nitric oxide are difficult to perform in young children. A rapid and straightforward diagnosis method for doctors is crucial if an adequate correlative biomarker can be identified. In our group, we revealed that IgE and TSLP levels in the peripheral blood of children with allergic asthma are higher than those in normal children. Furthermore, *TSLPR* mRNA expressions in the peripheral blood mononuclear cells of children with allergic asthma are significantly higher than those in other groups (nonallergic nonasthmatic, nonallergic asthma and allergic nonasthmatic) [103]. However, clarifying and identifying the regulation of TSLPR and IL-7Rα in pediatric asthma is still difficult because the type of blood cells and the type of expression for each blood cell in different stages of atopic diseases are poorly understood. We hypothesize that TSLPR heterocomplex expression by each peripheral blood cell subset is different in pediatric atopic asthma (Figure 2). Further investigation of the following issues is recommended: (1) the two isoform ratios of TSLP during atopic development and the therapeutic period; (2) TSLPR heterocomplex expression (inducible by allergic stimulation or hereditarily); and (3) the intra-structural stability of TSLPR heterocomplex and the affinity or avidity between the two isoforms of TSLPs and the TSLPR heterocomplex. We believe that an integrated assessment of these issues can provide a faster and earlier diagnosis for pediatric asthma and promote the development of more effective preventive strategies in the future.
